# Stiff-Person Syndrome: Diagnostic and Therapeutic Insights Into a Rare Autoimmune Neurological Disorder

**DOI:** 10.7759/cureus.96786

**Published:** 2025-11-13

**Authors:** Taoufik Boubga, Yahya Naji, Nawal Adali

**Affiliations:** 1 Department of Neurology, Military Hospital Oued Eddahab, Agadir, MAR; 2 Department of Neurology, Agadir Medical School, Ibn Zohr University, Agadir, MAR; 3 Department of Neurology, University Hospital of Agadir, Agadir, MAR; 4 Department of Neurology, Faculty of Medicine and Pharmacy of Agadir, Ibn Zohr University, Agadir, MAR

**Keywords:** anti-gad antibodies, autoimmune neurology, case report, intravenous immunoglobulin (ivig), rituximab, stiff-person syndrome

## Abstract

Stiff-person syndrome (SPS) is a rare autoimmune neurological disorder that is frequently underrecognized due to overlapping musculoskeletal and neuropsychiatric symptoms, leading to delays in diagnosis. We report a 56-year-old Moroccan woman with a seven-year history of progressive lumbar stiffness and painful, stimulus-sensitive spasms extending to both lower limbs, resulting in marked functional limitation and falls. Serum testing showed markedly elevated anti-glutamic acid decarboxylase (anti-GAD) antibodies (greater than 20,000 U/mL), while the spine MRI revealed only mild degenerative changes, and whole-body positron emission tomography excluded malignancy. Symptomatic therapy with pregabalin and baclofen provided partial relief. Intravenous immunoglobulin was administered at a total dose of 2 g/kg over five days, yielding a substantial reduction in stiffness and spasm frequency; at one month, she demonstrated improved mobility and greater independence. Rituximab was proposed for long-term disease control given disease severity and seropositivity. This case underscores the diagnostic value of anti-GAD testing when imaging is unrevealing and highlights that timely initiation of immunotherapy, together with symptomatic agents and rehabilitation, can meaningfully improve outcomes. A multidisciplinary strategy is essential for sustained functional recovery and quality of life in SPS.

## Introduction

Stiff-person syndrome (SPS) is an uncommon neurological disorder that poses unique challenges for both diagnosis and treatment. Although considered rare, its disabling nature and complex clinical presentation make it an important condition to investigate. The syndrome is characterized by progressive muscle stiffness and stimulus-sensitive spasms, symptoms that not only limit mobility but also severely impact quality of life. Because its clinical manifestations overlap with other neuromuscular and psychiatric conditions, SPS is frequently underrecognized or misdiagnosed, leading to delayed interventions.

Over the past several decades, advances in neuroimmunology have shed light on the autoimmune basis of SPS. The discovery of circulating autoantibodies against glutamic acid decarboxylase (GAD) and other neuronal proteins has been central to understanding its pathophysiology. Nevertheless, the precise mechanisms through which these immune responses disrupt inhibitory neurotransmission remain only partially understood. Furthermore, considerable heterogeneity exists in clinical phenotypes, antibody profiles, and responses to therapy, underscoring the complexity of this disorder.

Despite progress in immunotherapy and symptomatic management, SPS continues to be associated with significant morbidity, fluctuating treatment outcomes, and limited evidence-based guidelines. These gaps highlight the need for continued investigation into its epidemiology, immunological underpinnings, and therapeutic strategies. By deepening our understanding of SPS, research not only benefits those directly affected but also offers broader insights into the interplay between the immune system and the nervous system.

## Case presentation

The patient is a 56-year-old woman from Morocco, with no significant past medical history, who presented with longstanding lower back pain and progressive stiffness of the lower extremities. Her symptoms first appeared seven years ago as intermittent lumbar discomfort and mild rigidity, which initially improved with rest and analgesics. Over time, her symptoms progressed to constant lower back stiffness accompanied by painful spasms and reduced mobility. Approximately three years after onset, the stiffness extended to her thighs and calves, leading to progressive gait difficulties and frequent falls. Despite multiple consultations with her primary physician and trials of physical therapy, as well as over-the-counter medications, her symptoms continued to worsen, and her level of independence declined.

In the past year, the patient reported marked worsening of her symptoms, with severe stiffness in her lower limbs and spasms triggered by emotional stress and sudden noise. She described increasing difficulty standing upright, reliance on family members for mobility, and episodes of nocturnal pain disrupting sleep. She denied smoking, alcohol, or recreational drug use, and there was no family history of autoimmune or neurological disorders.

On admission, she was alert, oriented, and in no acute distress. Vital signs were within normal limits. Neurological examination revealed pronounced rigidity of the lumbar paraspinal muscles and proximal lower limbs. Attempted passive flexion of the hips and knees provoked painful spasms and was markedly limited. Upper extremities were unaffected, with normal tone, strength, and range of motion. Strength testing of the distal lower limbs revealed preserved dorsiflexion and plantarflexion, but assessment of proximal muscle power was limited by stiffness. Deep tendon reflexes were absent at the knees and ankles but normal in the upper extremities. Plantar responses were equivocal. Sensory and cranial nerve examinations were unremarkable. A summary of laboratory findings, along with reference ranges, is provided in Table 1.

**Table 1:** Laboratory findings of the patient TSH: thyroid-stimulating hormone; CSF: cerebrospinal fluid

Test	Result	Reference range
Erythrocyte sedimentation rate	Normal	0-20 mm/hour
Creatine kinase	280 U/L (mildly elevated)	30-200 U/L
Antiglutamic acid decarboxylase antibodies	>20,000 U/mL (positive)	<5 U/mL
Antinuclear antibody	Negative	Negative
Antineutrophil cytoplasmic antibodies	Negative	Negative
Rheumatoid factor	Negative	<20 IU/mL
Anti-SSA/SSB antibodies	Negative	Negative
Thyroid function tests (TSH, free T4)	Within normal limits	TSH: 0.4-4.0 mIU/L; free T4: 0.8-1.8 ng/dL
Cerebrospinal fluid protein	32 mg/dL (normal)	15-45 mg/dL
CSF glucose	65 mg/dL (normal)	50-80 mg/dL
CSF cell count	3 cells/µL	0-5 cells/µL

Initial laboratory evaluation showed normal erythrocyte sedimentation rate and no evidence of systemic inflammation. Serum creatine kinase was mildly elevated. The autoimmune panel was significant for strongly positive anti-GAD antibodies (greater than 20,000 U/mL). Other autoantibodies, including antinuclear antibody, antineutrophil cytoplasmic antibody, rheumatoid factor, anti-SSA, and anti-SSB, were negative. Thyroid function tests were within normal limits. Cerebrospinal fluid analysis was unremarkable, with normal protein and glucose and no pleocytosis.

Chromosomal analysis was not performed, as there is no known chromosomal abnormality associated with SPS. The diagnosis in this case was based on clinical features and the presence of high-titer anti-GAD antibodies, consistent with autoimmune etiology.

**Figure 1:**
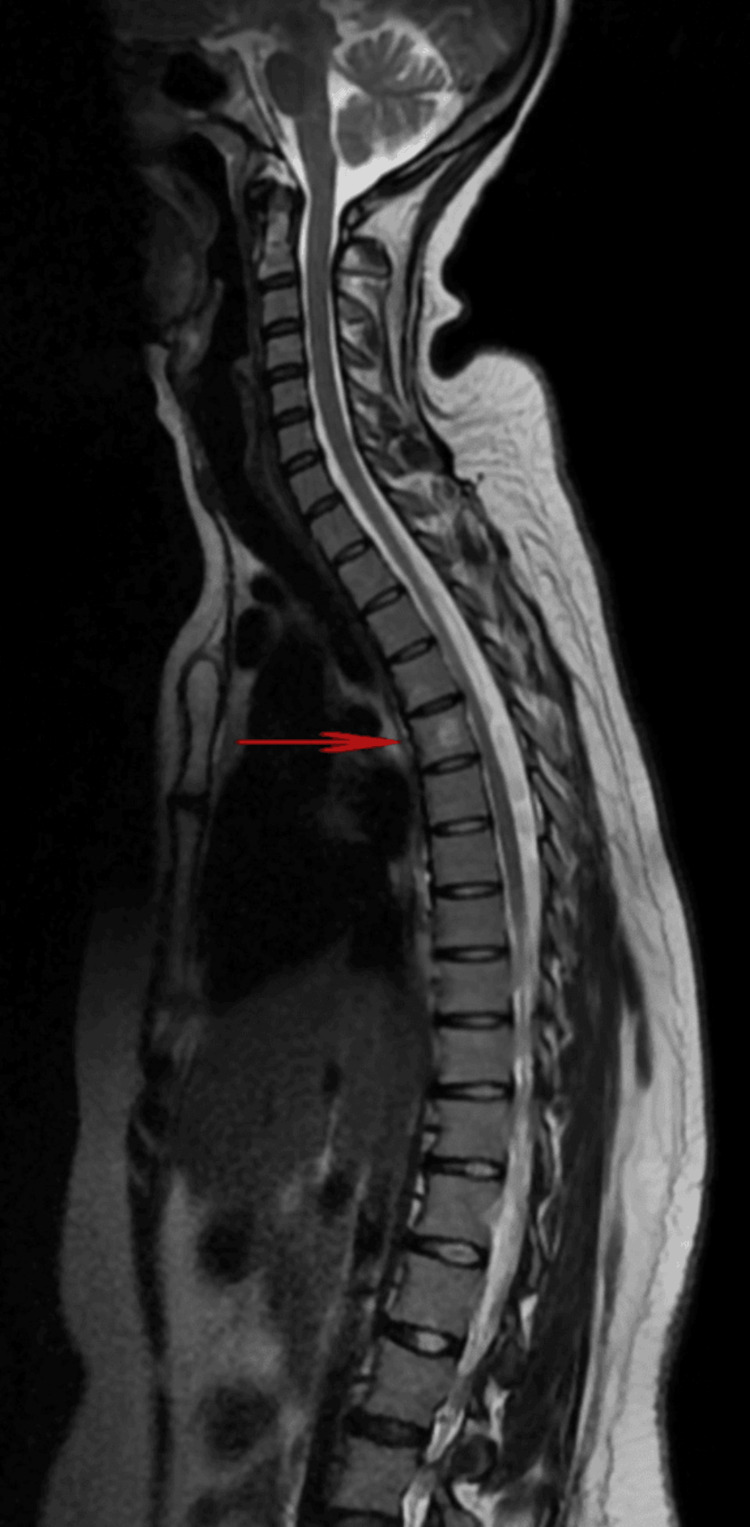
Sagittal T2 MRI of the spinal cord with mild degenerative changes

Neuroimaging included an MRI of the spine, which demonstrated only mild degenerative changes without evidence of demyelination, a mass lesion, or an inflammatory process (Figure 1). A whole-body positron emission tomography (PET) scan was normal, excluding occult malignancy.

The patient was treated with pregabalin and Lioresal (baclofen), which provided partial symptomatic relief. She subsequently received intravenous immunoglobulin (IVIG) at a total dose of 2 g/kg administered over five days, leading to a clear reduction in muscle stiffness and frequency of spasms. Rituximab therapy was proposed as part of her long-term management strategy, given the severity of her symptoms and strong anti-GAD antibody positivity.

At one-month follow-up, the patient demonstrated good improvement. She reported less rigidity, better mobility, and a reduction in spasms. She regained partial independence in her daily activities, including the ability to ambulate short distances with assistance. She remains on pregabalin and Lioresal, with scheduled monthly IVIG and planned initiation of rituximab therapy for sustained disease control.

## Discussion

SPS is a rare, chronic, and disabling neurological disorder with an estimated prevalence of one to two cases per million people worldwide [1]. Although initially described in 1956, awareness among clinicians remains limited, contributing to frequent delays in diagnosis [2]. The condition occurs more often in women, with most cases presenting between the ages of 30 and 60 years [3], and it is frequently associated with other autoimmune diseases such as type 1 diabetes mellitus, autoimmune thyroiditis, and pernicious anemia [4]. Variants of SPS have also been described, including partial forms restricted to the limbs, progressive encephalomyelitis with rigidity and myoclonus, and paraneoplastic forms, which are often associated with amphiphysin or glycine receptor antibodies [5]. Our patient represents the classic form of SPS, with insidious onset, progressive stiffness, and confirmed anti-GAD antibody positivity, but without paraneoplastic association.

The pathophysiology of SPS centers on impaired inhibitory neurotransmission in the central nervous system. Anti-GAD antibodies, detected in approximately 60%-80% of cases, target the enzyme GAD, which is essential for the synthesis of γ-aminobutyric acid (GABA) [6]. The resulting reduction in GABAergic activity leads to excessive firing of motor neurons and persistent muscle contractions. In some patients, alternative antibodies, such as those against amphiphysin or glycine receptors, suggest different immunological mechanisms and may define specific disease subtypes. In our case, the markedly elevated anti-GAD titers (greater than 20,000 U/mL) strongly supported the diagnosis and provided a clear mechanistic link to the clinical manifestations.

Diagnosing SPS is often challenging. Patients may present initially with vague musculoskeletal complaints, such as chronic low back pain or stiffness, which are frequently misattributed to degenerative or rheumatologic conditions. Psychiatric misdiagnoses, including functional movement disorder or somatoform disorder, are also common due to the unusual nature of spasms and their sensitivity to emotional triggers [7]. Neuroimaging typically demonstrates nonspecific or absent findings, as in our patient, whose MRI revealed only mild degenerative changes. The absence of clear radiological correlates underscores the importance of antibody testing, particularly for anti-GAD, in guiding diagnosis [8]. Whole-body PET scanning, which was normal in this patient, is often indicated to exclude paraneoplastic syndromes in individuals with atypical antibody profiles or systemic symptoms.

The management of SPS requires a multimodal approach that addresses both symptomatic control and immunomodulation. Symptomatic therapy aims to enhance GABAergic tone through agents such as benzodiazepines (diazepam, clonazepam), baclofen, gabapentin, or pregabalin [9]. Our patient received pregabalin and Lioresal (baclofen), which offered partial symptomatic relief. However, disease progression necessitated immunotherapy. IVIG, administered in a standard 2 g/kg regimen over five days, is one of the best supported treatments and has been shown in randomized controlled trials to reduce stiffness scores and improve mobility [10]. The marked improvement in our patient following IVIG is consistent with these findings.

Rituximab, a monoclonal antibody targeting CD20-positive B cells, has been increasingly used in refractory cases, with case series and observational studies suggesting clinical benefit, though randomized data remain inconclusive [11]. Other immunomodulatory therapies include corticosteroids, plasma exchange, mycophenolate mofetil, and cyclophosphamide, though their efficacy is variable and limited by side effects. In recent years, novel strategies such as stem cell transplantation and targeted biologics have been explored, but these remain investigational. Our patient's treatment plan, which includes continuation of IVIG and the proposal of rituximab, reflects current practice aimed at achieving long-term disease stabilization.

Prognosis in SPS is highly variable. Some patients respond well to therapy and maintain functional independence, while others experience progressive disability despite aggressive treatment. The most disabling features include severe rigidity, recurrent falls, and dependence on caregivers for daily activities. Psychiatric comorbidities such as anxiety and phobias are common and further impair quality of life. Our patient's favorable short-term response, with good improvement at one-month follow-up, is encouraging, but long-term monitoring is essential given the chronic and relapsing nature of the disease.

This case highlights several key learning points. First, SPS may present with a long prodromal period of nonspecific symptoms, such as low back pain, before more characteristic features emerge. Clinicians should maintain a high index of suspicion in patients with unexplained stiffness and spasms, particularly when routine imaging is unrevealing. Second, serological testing for anti-GAD antibodies remains a cornerstone of diagnosis, distinguishing SPS from mimics and guiding treatment decisions [2]. Third, early initiation of immunotherapy, particularly IVIG, can lead to meaningful improvement even in patients with advanced disease duration [10]. Ultimately, long-term disease management necessitates a multidisciplinary approach that combines pharmacological therapy, physical rehabilitation, and psychosocial support.

## Conclusions

SPS is a rare but disabling autoimmune neurological disorder that often presents with insidious and nonspecific symptoms, leading to delays in recognition and treatment. Our case emphasizes the importance of maintaining clinical suspicion in patients with progressive lower back pain and stiffness unresponsive to conventional therapies. The detection of high anti-GAD antibody titers remains central to confirming the diagnosis. Early initiation of immunotherapy, particularly IVIG, can significantly improve symptoms and restore functional independence, even in patients with longstanding disease. Long-term management requires a multidisciplinary approach, with immunomodulatory therapies such as rituximab offering additional potential for sustained disease control. Timely diagnosis and treatment are essential to improving outcomes and quality of life in affected patients.
